# Genome-wide association studies reveal candidate genes associated to bacteraemia caused by ST93-IV CA-MRSA

**DOI:** 10.1186/s12864-021-07738-4

**Published:** 2021-06-05

**Authors:** Stanley Pang, Denise A Daley, Shafi Sahibzada, Shakeel Mowlaboccus, Marc Stegger, Geoffrey W Coombs

**Affiliations:** 1 Antimicrobial Resistance and Infectious Diseases (AMRID) Research Laboratory, Murdoch University, Murdoch, Western Australia Australia; 2 Department of Microbiology, PathWest Laboratory Medicine-WA, Fiona Stanley Hospital, Murdoch, Western Australia Australia; 3 Australian Group on Antimicrobial Resistance (AGAR), Fiona Stanley Hospital, Murdoch, Australia; 4 Department of Bacteria, Parasites and Fungi, Statens Serum Institut, Copenhagen, Denmark

**Keywords:** *Staphylococcus aureus*, GWAS, Bacteraemia, Phylogenomics, Australia

## Abstract

**Background:**

The global emergence of community-associated methicillin-resistant *Staphylococcus aureus* (CA-MRSA) has seen the dominance of specific clones in different regions around the world with the PVL-positive ST93-IV as the predominant CA-MRSA clone in Australia. In this study we applied a genome-wide association study (GWAS) approach on a collection of Australian ST93-IV MRSA genomes to screen for genetic traits that might have assisted the ongoing transmission of ST93-IV in Australia. We also compared the genomes of ST93-IV bacteraemia and non-bacteraemia isolates to search for potential virulence genes associated with bacteraemia.

**Results:**

Based on single nucleotide polymorphism phylogenetics we revealed two distinct ST93-IV clades circulating concurrently in Australia. One of the clades contained isolates primarily isolated in the northern regions of Australia whilst isolates in the second clade were distributed across the country. Analyses of the ST93-IV genome plasticity over a 15-year period (2002–2017) revealed an observed gain in accessory genes amongst the clone’s population. GWAS analysis on the bacteraemia isolates identified two gene candidates that have previously been associated to this kind of infection.

**Conclusions:**

Although this hypothesis was not tested here, it is possible that the emergence of a ST93-IV clade containing additional virulence genes might be related to the high prevalence of ST93-IV infections amongst the indigenous population living in the northern regions of Australia. More importantly, our data also demonstrated that GWAS can reveal candidate genes for further investigations on the pathogenesis and evolution of MRSA strains within a same lineage.

**Supplementary Information:**

The online version contains supplementary material available at 10.1186/s12864-021-07738-4.

## Background

Over the last three decades, community-associated methicillin-resistant *Staphylococcus aureus* (CA-MRSA) has emerged globally. Although polyclonal, a small number of CA-MRSA clones are dominant in different regions of the world such as multilocus sequence type (ST) 8-IV (USA300) in North America, ST80-IV in Europe and Northern Africa, ST59-IV/V in Asia, ST772-V and ST22-IV in the Indian subcontinent, and ST30-IV in the West Pacific region [1]. Transmission of the dominant clones in other regions has occurred, and characteristically they harbour the *lukS/F-PV* genes that encode the Panton-Valentine leukocidin (PVL) toxin [2].

In Australia, the dominant CA-MRSA clone is PVL-positive ST93-IV[3]. Colloquially known as the “Queensland CA-MRSA clone”, ST93-IV was first described in the early 2000 s. Although known to cause severe infections including necrotizing pneumonia, ST93-IV is typically associated with skin and soft tissue infections [4]. Reported across Australia, the clone is frequently isolated in the indigenous Australian population where its dominance is believed to be linked to overcrowding [5], poor hygiene and healthcare [6]. Using whole genome sequencing (WGS) and temporal and geographical analysis, ST93 has been shown to be an early diverging and recombinant lineage genetically related to ST59/ST121 and to an unknown *S. aureus* lineage that emerged in the 1970 s in the North Western region of Australia [5]. Although earlier studies into the genetic diversity of ST93 showed multiple rearrangements of the *spa* sequence, the core regions of the genome were very stable [2]. However in 2014, Stinear et al. suggested ST93 clone was under pressure for adaptive change due to a reduction in both exotoxin expression and oxacillin minimum inhibitory concentration [7].

To screen for potential association between gene content and disease, genome-wide association studies (GWAS) can be performed by analysing single nucleotide polymorphisms (SNPs), and the accessory genes provided by WGS data. For example, GWAS performed on isolates from children with acute *S. aureus* osteomyelitis selected a number of virulence gene candidates potentially associated to the severity of disease [8]. In contrast, when applied to *S. aureus* bacteraemia isolates, no obvious associations in the number of virulence genes present in isolates from patients with and without *S. aureus* infective endocarditis were identified [9]. GWAS can also be used to examine the evolution of a bacterial clone. For example recent GWAS performed on livestock-associated CC398 MRSA, showed the clone frequently lost antimicrobial resistance genes and acquired human specific virulence genes in relation to the origin of the host [10].

In this study, we performed GWAS on a collection of Australian ST93 MRSA bacteraemia isolates collected over a three-year period (2015–2017) and a collection of previously published ST93 MRSA genomes (2002–2012). Phylogenetic analysis of the genomes was performed by examining SNPs in the core genome and investigating the absence/presence of accessory genes. To screen for potential genetic traits that may have assisted the ongoing transmission of ST93-IV in Australia we correlated the absence and presence of accessory genes in the ST93-IV genomes to time, location and whether they originated from a bloodstream infection.

## Results

The 423 ST93-IV were isolated across Australia from the following states and mainland territories: Northern Territory (n = 141), Queensland (n = 98), New South Wales (n = 64), Western Australia (n = 54), Victoria (n = 43), South Australia (n = 19), Australia Capital Territory (n = 3) and Tasmania (n = 1). Overall, there were 302 bacteraemia and 121 non-bacteraemia isolates. The non-bacteraemia isolates were limited to four geographical regions: New South Wales, Victoria, Western Australia and Northern Territory.

**Fig. 1:**
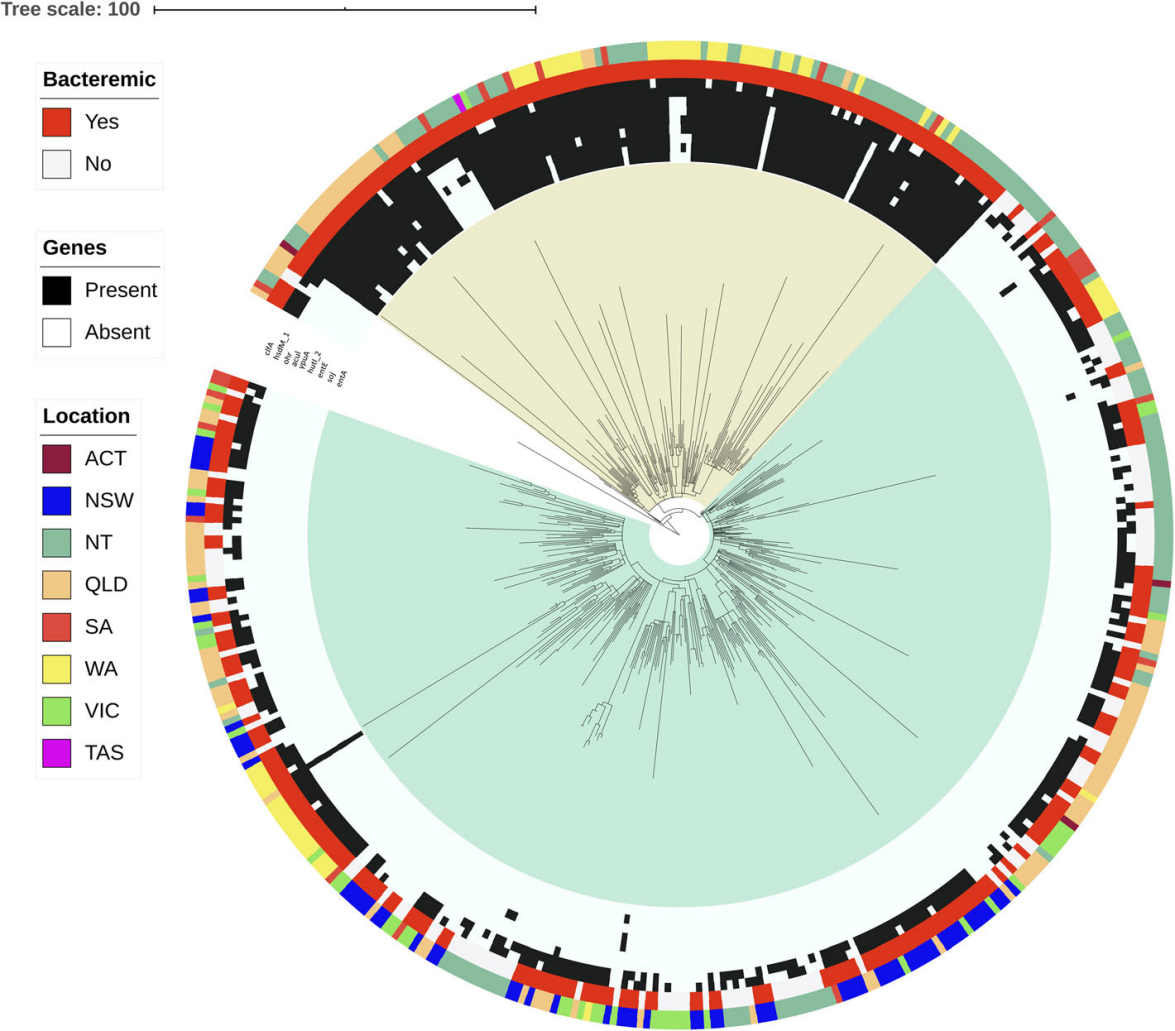
Rooted Phylogenetic tree of 423 ST93 *S. aureus* bacteraemia and non-bacteraemia genomes represented as red and white respectively (outer ring). Location is represented by the abbreviation of Australian states and territories: Australian Capital Territory (ACT), New South Wales (NSW), Northern Territory (NT), Queensland (Qld), South Australia (SA), Western Australia (WA), Victoria (Vic) and Tasmania (Tas). Genes present (black) and absence (grey) that correlate with bacteraemia are listed in the order (outer to inner); *clfA, hsdM_1, ohrR, acuI, ypuA, hutl_2, entE, soj* and *entA_2*

Based on core genome SNPs, the rooted phylogeny based on 1383 SNPs depicted the ST93 population to cluster primarily in two main clades (Fig. 1). Clade 1 contained 111 bacteraemia isolates predominantly from northern Australia whilst clade 2 contained 185 bacteraemia and 119 non-bacteraemia isolates collected across Australia.

### Comparison between Principal Component Analysis (PCA) and Phylogenetic Clustering

**Fig. 2:**
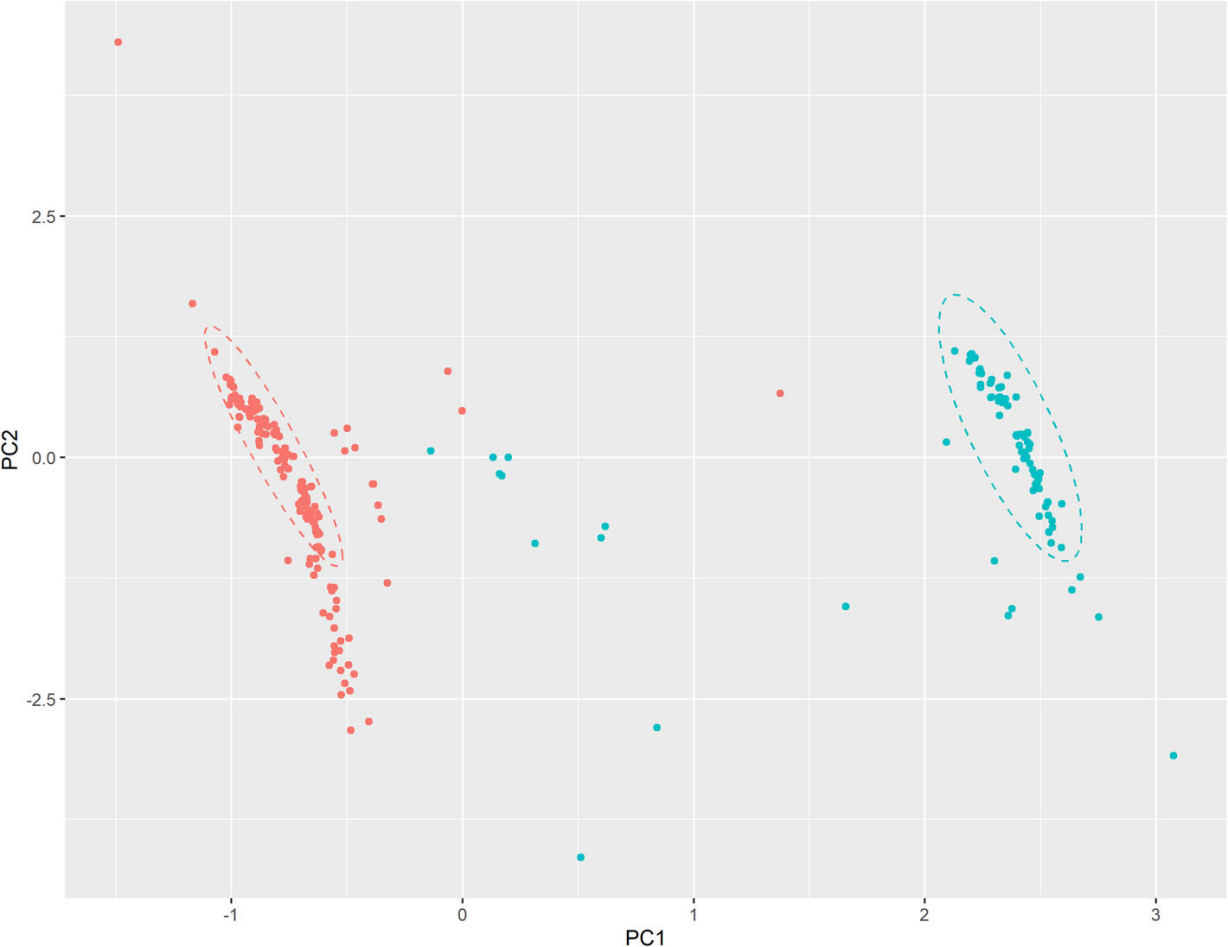
Principal Component Analysis of pan-genome gene matrix of ST93-IV isolates. The teal coloured dots represent isolates in clade 1, while the red coloured dots represent isolates in clade 2. Non-clade 1 and 2 isolates are grey coloured dots. The ellipse is generated using the multivariate t- distribution with CI = 95 %

By examining the presence and absence of accessory genes, PCA identified two distinct clusters (Fig. 2). Isolates in the two PCA clusters correlated with isolates in the two SNP derived phylogenetic clades.

### GWAS Comparison between Bacteraemia and Non-bacteraemia ST93 Isolates

GWAS revealed nine accessory genes correlated with the bacteremia isolates (*p* < 0.001 and odds ratio > 1) (Table 1). However, seven of these genes were clade 1 specific and were not considered bacteraemia factors (Supplementary Table 2).

**Table 1:** GWAS showing genes significantly correlating to bacteraemia using the presence (+) and absence (-) of each gene in 423 isolates (Bonferroni p value < 0.001 and a odds ratio > 1), * genes specific to Clade 1

Gene	Function	Bacteraemia Isolates N (%)	Non Bacteraemia Isolates N (%)
*clfA*	Clumping factor A	240 (79.4)	53 (43.8)
*hsdM_1*	Type I restriction enzyme EcoKI M protein	269 (89)	55 (45)
*ohrR*	Organic hydroperoxide resistance transcriptional regulator*	103 (34.1)	0
*acul*	Putative acrylyl-CoA reductase Acul*	102 (33.7)	0
*ypuA*	Hypothetical protein*	105 (34.7)	1 (0.7)
*hutl_2*	Hypothetical protein*	101 (33.4)	1 (0.7)
*entE*	Enterotoxin type E*	101 (33.4)	0
*soj*	Chromosome partitioning ATPase*	101 (33.4)	1 (0.7)
*entA_2*	Enterotoxin type A*	99 (32.7)	0

Because the majority of clade 1 genomes were bacteremia isolates, GWAS was repeated without clade 1 genomes to remove a possible selection bias. The results for both GWAS showed that the two genes that correlated with bacteraemia were *hsdM* (type I restriction enzyme *Eco*KI M protein) and *clfA* (clumping factor A) (Supplementary Table 3). Overall, of the 302 bacteraemia isolates, 76 % (n = 230) carried both genes; 16 % (n = 49) carried one of the genes, and the remaining 7 % (n = 23) carried neither gene. Only 43 and 45 % of the non-bacteraemia isolates carried the *clfA* and *hsdM* genes respectively.

The seven clade 1 specific accessory genes were o*hrR* (organic hydroperoxide resistance transcriptional regulator), *acul* (putative acrylyl-Coa reductase), *ypuA* (hypothetical protein), *hutl_2* (hypothetical protein), *entE* (enterotoxin E), *soj* (chromosome-partitioning ATPase) and e*ntA_2* (enterotoxin A) (Fig. 1). Approximately 88 % (*n* = 98/111) of the clade 1 genomes harboured all seven genes, with seven isolates containing none of the seven genes. The seven genes were located on five different contigs, with *entE* and *acuI* co-located with *soj* and *oh*R respectively.

### Genomic diversity of ST93 over Time and Location

No significant differences in the presence or absence of accessory genes over time or location were identified.

### Recombination/rearrangement of the ST93 genome

When we analysed conserved gene neighbourhoods, we observed two genes affected by re-arrangements correlating to bacteraemia, *sdrF* (serine-aspartate repeat-containing protein F) and *pls* (surface protein) (Supplementary Table 4). Analysis of the genes show that inversions occurred in regions containing *sdrF* and *pls* (Supplementary Figure 1).

## Discussion

In the current study we have identified two distinct ST93-IV clades circulating concurrently in Australia. The identification of the two clades by SNP analysis of the core region was supported by the PCA based on the absence and presence of genes matrix. The clade 1 isolates were primarily isolated in the northern regions of Australia spread over three states/territories (Western Australia, Northern Territory and Queensland) whilst the clade 2 isolates were distributed across the country. Based on genomic data of the van Hal et al. [5] historic ST93-IV isolates that were located at the root of the phylogenetic tree, we believe the two clades recently diverged from a common ancestor.

Clade 1 isolates differed from the clade 2 isolates by having acquired up to seven additional accessory genes. The known biological significance of these accessory genes varies. The *entA* and *entE* genes, encode the superantigen enterotoxins A and E respectively and play an important role in serious staphylococcal infections by triggering an overexpression of inflammatory mediators [11]. The *ohrR* gene, which has previously been identified in *Pseudomonas aeruginosa* [12] and *Bacillus subtilis* [13], is known to increase an organism’s resistance to oxidative stress. The ability to resist peroxide provides the organism a growth advantage and increases its survival in host cells [14]. The s*oj* gene, a *parA* homologue involved in chromosome segregation during DNA replication, is not normally found in *S. aureus* [15]. Typically, chromosome segregation in *S. aureus* is performed by the *parB* homologue s*po0J*, which was identified in all ST93-IV genomes. In *Bacillus subtilis*, *soj* and *spo0J* are present and work together to prevent premature midcell Z ring assembly [16]. By having acquired *soj*, clade 1 isolates might have an advantage over non-clade 1 isolates as represented by a more efficient DNA replication system. The roles of the three remaining accessory genes, *acul* (a putative protein), and *ypuA* and *hutl* (both hypothetical proteins) are not known. The acquisition of the seven accessory genes, which are likely to have originated on mobile genetic elements, may explain the high rates of ST93-IV skin infections amongst indigenous children living in the northern regions of Australia [17]. Further studies are required to determine if clade 1 has become the predominant ST93-IV strain in the region’s indigenous communities and the role of these additional genes in the expansion and fitness of this pathogen.

Based on the variability of the ST93-IV accessory genes over time and location we attempted to identify clade 1 or 2 specific subclades. Despite minor accessory gene variations occurred in a small number of isolates (for example, four isolates contained *qacA* [antiseptic resistance protein], *qacR* [HTH-type transcriptional regulator] and *tnsB* [transposon] which were all located on the same contig), no important difference in the absence or presence of accessory genes related to specific subclades were observed.

### GWAS for Bacteraemia vs. Non-bacteraemia MRSA

In 2017 a GWAS performed by Lilje and colleagues was not able to identify genetic differences between *S. aureus* bacteraemia and non-bacteraemia genomes [9]. Their results however may have been influenced by studying a variety of *S. aureus* lineages and clonal complexes. To identify if specific genetic factors are harboured by *S. aureus* bacteraemia genomes our study was limited to a single *S. aureus* lineage. After accounting for a possible clade 1 selection bias GWAS identified two genes associated with the ST93-IV bacteraemia isolates. The *hsdM* gene has recently been shown to be a hotspot for chromosome rearrangements in staphylococcus which cause phenotype switching associated with persistent infections [18]. The *clfA* gene, which mediates staphylococcal binding to fibrin-coated surfaces has previously shown to be highly expressed during rat models in infective endocarditis [19], while *clfA* mutants developed milder systemic inflammation in mice models [20].

Chromosome rearrangements of genes may lead to altered gene expression [21]. The 23 bacteraemic genomes that did not harbor *hsdM* and *clfA* all carried rearrangements of the *pls* and *sdrF*, genes. The *pls* and *sdrF* genes encode surface proteins. Pls, which mediates bacterial aggregation and binding to glycolipids and human epithelial cells [22, 23], has been shown in mice models to be an important factor in causing sepsis [24]. SdrF, which is a microbial surface components recognising adhesive matrix molecule (MSCRAMM), allows staphylococcus to attach to and colonise host cells [25]. Among the *sdr* gene detected amongst the different *S. aureus* clones, the *sdr* gene in the ST93 strain JKD6159 is the most diverse suggesting *sdr* acquisition by horizontal gene transfer. In the Huping et al. study the *sdr* in the ST93 genome was classified as *sdrC* [26]. However, an updated annotation database has identified the gene as *sdrF* which had previously only been reported in *S. epidermidis.* SdrF adheres to human keratinocytes and epithelial cells facilitating *S. epidermidis* colonisation of the skin [27].

## Conclusions

GWAS is a powerful tool to screen for potential associations using large datasets. However, other factors related to bacterium-host evolution may also pressure for genetic diversification. For example, patient’s age and prior medical condition, which are factors associated with MRSA bacteraemia. In the current study we selected accessory genes and gene rearrangements that show significant statistical associations with ST93-IV bacteraemia. However, to validate the scientific impact of these findings future ex vivo and in vivo investigations using gene-knockouts and expression clones are required. Furthermore, to determine if these genes are bacteraemia determinants other *S. aureus* lineages should be examined. Finally, phylogenetic analysis has shown ST93-IV has recently gained accessory virulence genes which might be contributing to the clone’s persistence in the Australian indigenous communities.

## Methods

### Bacterial Strains and Genome Assembly

A total of 300 ST93 MRSA bacteraemia isolates were identified in the 2015 [28], 2016 [29] and 2017 [30] Australian Group on Antimicrobial Resistance (AGAR) Australian *Staphylococcus aureus* Sepsis Outcome Programs (ASSOPs). All isolates collected were from patients with systemic infections. As part of ASSOP, MRSA isolates were referred to a central reference laboratory where genomic libraries were prepared using the Illumina Nextera^®^ XT DNA Library Prep Kit (Illumina, United States) according to the manufacturer’s protocol. WGS was performed on the Miseq or Nextseq platforms using the Miseq Reagent Kit V3 (600 cycle) and the Nextseq 500/550 Mid Output Kit V2.5 (300 cycles), respectively. The raw sequence reads were assembled *de novo* using SPAdes V3.12 [31]. Sequencing quality control was determined based on average sequencing depth. Thirty-one had genomes less with than 40x coverage and therefore were excluded. The MLST profiles of the remaining 269 genomes were determined using the mlst tool described by Seeman et al. [32].

In addition to the 269 ASSOP ST93 MRSA, whole genome sequences for 154 ST93 MRSA collected between 2002 and 2012 from Van Hal et al. study [5] were included (Supplementary Table 1).

All sequence data obtained from this study were deposited to the NCBI Sequence Read Archive under BioProject ID PRJNA644215.

### Phylogenetic Analysis

Using the chromosome of *S. aureus* CC398 reference strain SO395 (GenBank accession ID AM990992) as the reference genome, the bacterial variant calling tool snippy V4.1.0 [33] was used to extract and align SNPs from the core genome. The 423 ST93 genomes were used to generate a rooted maximum parsimony phylogenetic tree using MEGA V10.1.7 [34] with the following parameters; bootstrap value: 1000, nucleotide substitution model and the SPR model for the MP search method. Phylogenetic clades were defined as a cluster of isolates sharing multiple common SNP mutations. The iTOL V3 web service was used to visualise the phylogenetic tree and the corresponding metadata [35].

### Genome-Wide Association Study (GWAS)

Genes from the 423 assembled *S. aureus* genome sequences were annotated with Prokka V1.13 [36] using default parameters and the pan-genome was extracted by Roary V3.12.0 [37] using the -s option of no paralog splitting. The pan-genome matrix from Roary containing of gene presence or absence for each genome was used as input for Scoary V1.6.16 [38] with the following traits; SNP phylogeny clades, location (states and territories), year of isolation, clade specific genes and whether the isolate was from a bloodstream infection. Adapting the method described by Arnoud H. M. van Vliet [39], genes returning a Bonferroni corrected *p* value ≤ 10^− 5^ and odds ratio > 1 were further investigated. In addition to Scoary analysis, principal component analysis (PCA) of binomial variables on the pan-genome matrix was performed for determination of association with the statistical package R version 3.5.1 [40] and ggplot2 V3.2.1 to confirm relationships between genes identified in Scoary and traits.

### Detecting gene rearrangements

The pan-genome matrix was compared with and without the -s option. Scoary was used on both pan-genome matrices using bacteremia as phenotype. Identification of genes correlating to bacteremia were compared between both sets of data. Genes associated to bacteremia were extracted along with neighbouring genes and aligned against the ST93 genome JDK6159 using Artemis comparison tool [41] to visualise the rearrangement structure.

## Supplementary Information

**Additional file 1:Supplementary Table 1**: ST93-IV isolates used in this study with bacteremia gene matrix

**Additional file 2:Supplementary Table 2**: Statistics supporting clade specific genes

**Additional file 3:Supplementary Table 3**: Statistics supporting bacteremia in clade 2

**Additional file 4:Supplementary Table 4**: Gene rearrangements statistics associated to bacteremia

**Additional file 5:Supplementary Figure 1**: Rearrangements of A) pls and B) sdrF gene neighbourhoods.

## Data Availability

The data that support the findings of this study are openly available on the SRA database under Bioproject: PRJNA644215, Accession: SRX8689588-SRX8689856. Collection of ST93 *S. aureus* used as reference in this study is available on ENA (Supplementary Table 1). An additional reference genome used is also available from GenBank (Accession: AM990992).

## Abbreviations

AGAR: Australian Group on Antimicrobial Resistance
ASSOPs: Australian *Staphylococcus aureus* Sepsis Outcome Programs
CA-MRSA: Community-Associated Methicillin-Resistant *Staphylococcus aureus*
CC: Clonal Complex
DNA: deoxyribonucleic acid
GWAS: Genome Wide Association Study
MSCRAMM: microbial surface components recognising adhesive matrix molecule
MLST: Multi Locus Sequence Typing
PCA: Principal Component Analysis
PVL: Panton-Valentine Leukocidin
SNP: Single Nucleotide Polymorphisms
ST: Sequence Type
WGS: Whole Genome Sequencing
