# Supplementary figures and images for: Genome-wide association studies reveal candidate genes associated to bacteraemia caused by ST93-IV CA-MRSA
Source: BMC Genomics. 2021 Jun 5;22:418. doi: 10.1186/s12864-021-07738-4 (PMC8180019; doi:10.1186/s12864-021-07738-4)

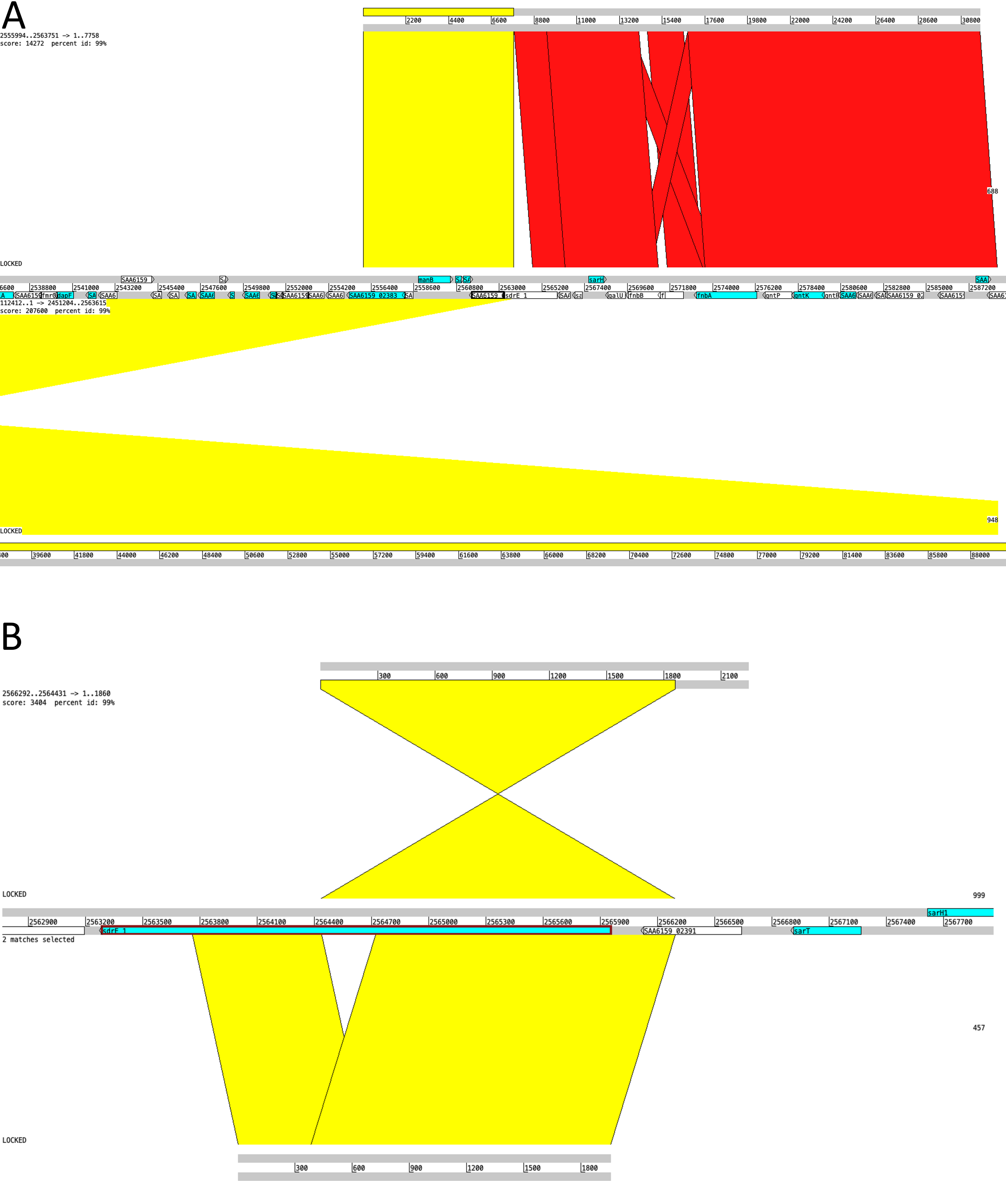

Supplement: Supplementary file 5 — Additional file 5:Supplementary Figure 1: Rearrangements of A) pls and B) sdrF gene neighbourhoods. [file 12864_2021_7738_MOESM5_ESM.png]
